# Natural Radioactivity in Raw Building Materials for Underground Parking Lots and Assessment of Radiological Health Risk for the Population

**DOI:** 10.3390/ijerph21030315

**Published:** 2024-03-08

**Authors:** Francesco Caridi, Giuseppe Paladini, Antonio Francesco Mottese, Filippo Giammaria Praticò, Giuliana Faggio, Giacomo Messina, Alberto Belvedere, Santina Marguccio, Maurizio D’Agostino, Domenico Majolino, Valentina Venuti

**Affiliations:** 1 Dipartimento di Scienze Matematiche e Informatiche, Scienze Fisiche e Scienze della Terra, Università degli Studi di Messina, V.le F. Stagno D’Alcontres, 31-98166 Messina, Italy; amottese@unime.it (A.F.M.); dmajolino@unime.it (D.M.); vvenuti@unime.it (V.V.); 2 Dipartimento di Fisica e Astronomia “Ettore Majorana”, Università degli Studi di Catania, Via S. Sofia, 64-95123 Catania, Italy; giuseppe.paladini@dfa.unict.it; 3 Dipartimento di Ingegneria dell’Informazione, delle Infrastrutture e dell’Energia Sostenibile (DIIES), Università “Mediterranea”, Loc. Feo di Vito, 89122 Reggio Calabria, Italy; filippo.pratico@unirc.it (F.G.P.); gfaggio@unirc.it (G.F.); messina@unirc.it (G.M.); 4 Agenzia Regionale per la Protezione dell’Ambiente della Calabria (ARPACal), Dipartimento di Reggio Calabria, Via Troncovito SNC, 89135 Reggio Calabria, Italy; a.belvedere@arpacal.it (A.B.); s.marguccio@arpacal.it (S.M.); m.dagostino@arpacal.it (M.D.)

**Keywords:** building materials, underground parking lots, natural radioactivity, radiological health risk, multivariate statistics, Pearson’s correlation, principal component analysis, hierarchical cluster analysis

## Abstract

This article reports the results of an investigation into the activity concentration of natural radionuclides in raw building materials for underground parking lots, together with the assessment of the radiation hazard for the public related to exposure to ionizing radiations. To this purpose, high-purity germanium (HPGe) γ-ray spectrometry was employed in order to quantify the average specific activity of ^226^Ra, ^232^Th, and ^40^K natural radioisotopes. With the aim to assess any possible radiological health risk for the population, the absorbed γ-dose rate (*D*), the annual effective dose equivalent outdoor (*AEDE_out_*) and indoor (*AEDE_in_*), the activity concentration index (*I*), and the alpha index (*I_α_*) were also estimated, resulting in values that were lower than the maximum recommended ones for humans. Finally, the extent of the correlations existing between the observed radioactivity and radiological parameters and of these parameters with the analyzed samples was quantified through statistical analyses, including Pearson’s correlation, a principal component analysis (PCA), and a hierarchical cluster analysis (HCA). As a result, three clusters of the investigated samples were recognized based on their chemical composition and mineralogical nature. Noteworthily, this paper covers a certain gap in science since its topic does not appear in literature in this form. Thus, the authors underline the importance of this work to global knowledge in the environmental research and public health fields.

## 1. Introduction

Natural radioactivity is a widespread phenomenon on a global scale due to the natural decay of primordial radionuclides, such as ^232^Th, ^238^U, and ^40^K, present in the Earth’s crust [1]. In particular, ^226^Ra, ^232^Th, and ^40^K present in building materials are important sources of human exposure [2]. Gamma radiation from these natural radioisotopes results in external radiation exposure. Building materials may be radioactive for several different reasons, mainly because of the raw materials with elevated activity concentrations of natural radioisotopes used in their manufacturing. It is, therefore, critical to continuously monitor the specific activity of radionuclides in construction materials. One of the approaches used to decrease the external exposure dose is to select materials with a low level of radionuclide activity. The evaluation of doses due to radioisotopes in construction materials is significant from the point of view of radiological safety. The high activity of natural radionuclides in construction materials can account for higher dose rates in indoor areas, particularly when products from a variety of manufacturers are utilized in production operations [3]. Since human beings are continually exposed to natural radiation, which is the most significant contributor to the external dose of the public [4,5], the evaluation of the gamma radiation dose, which is naturally originating, is of pivotal relevance for health care.

In this context, radon exhalation is also a major concern, as this radioactive inert gas is the most significant source of internal exposure. According to the literature [6], approximately 50% of the total dose from natural radiation should be ascribed to radon and its decay products. Although the main source of radon in indoor environments is soil, in some cases, the major sources of indoor radon can be construction materials [7]. In particular, radon is an emitter of alpha particles, and thus, its radiation is readily absorbed by human skin. Nevertheless, radon gas can also be inhaled by human beings, and its decay products (^218^Po, ^214^Pb, and ^214^Bi) can result in internal exposure [8]. The main risk posed by radon and its derivatives is that they can induce lung cancer. Radon dissipates quickly outdoors and is not a health concern. The majority of radon exposure happens in indoor environments, where this gas may increase to high concentration values. It was estimated that the equivalent dose rate due to ionizing radiation from the radon daughters ^214^Pb and ^214^Bi ranges from 2% to 20% of the overall equivalent dose rate [9]. The mean annual effective dose from natural sources is 2.4 mSv; of this value, 1.1 mSv is due to basic background radiation, and 1.3 mSv is due to radon exposure [10]. According to Italian legislation, the indoor radon-specific activity must be lower than 300 Bq m^−3^ [11].

For all the aforementioned reasons, the evaluation of human exposure to ionizing radiation has attracted growing attention from the scientific community, as can be seen by the increasing number of scientific papers related to the field [12,13,14,15]. In fact, there is an increasing awareness that radiation effects are induced by radioactive elements [16,17,18], whose distribution is not homogeneous over the Earth’s crust. As it is well known, long-term exposure to uranium and radium induces several health effects, including chronic lung diseases, acute leucopenia, anemia, and mouth necrosis. In particular, thorium exposure can cause lung, pancreas, hepatic, bone, and kidney cancers and leukemia [19]. As a consequence, several studies on natural radioactivity content were carried out on different environmental matrices [20,21,22,23]. Among them, raw building materials for underground parking lots, i.e., mixtures of rock, mineral, and glass particles, are regarded as promising naturally occurring resources for producing inexpensive and environmentally friendly construction materials with adequate resistance and durability properties, thus strongly contributing to sustainable growth [24,25]. Asphalt, the predominant surface layer in road construction, is also frequently used as a paving material in both car parks and underground garages. Drivable surfaces must withstand particular stresses, e.g., due to the influence of moisture or mechanical abrasion, and a good grip is also especially important in the entry and exit areas or on narrow and steep ramps [26]. Exposure to radon progeny concentration in underground parking garages is principally affected by the amount of ventilation and the emanation of radon from soil and construction materials. To lower the extent of this radioactive inert gas in carparks, it is important to employ means to block its emanation from building materials, as well as to learn about and monitor the specific activity of ^226^Ra in soil and construction materials, since the extent of radon exhalation depends on it [27]. It has been evaluated that the amount of construction material used and its specific location in a building determines the exposure [28]. To ensure that the public is exposed to a lower level of radiation from radioisotopes in building materials, the specific activity of radionuclides in construction materials must not exceed predefined threshold values. Noteworthily, after assessing the ionizing radiation exposure of radionuclides in construction materials and the variation and formation of that exposure, activities can be planned to minimize exposure on an optimal level. In fact, even if the usual amount of time people spend in underground garages ranges from minutes to several hours per day, there are some examples of exceptions. In Italy, for example, underground malls are home to car washing facilities, wheel services, and several small repair shops. Then, workers and customers typically spend a significant quantity of time in them. In addition, sports and training structures and alternative workshops can be set up in the underground parking lots of residential buildings. Thus, it is necessary to consider that some people are likely to spend a considerable amount of time in underground garages than other individuals. This is important from the point of view of radiation protection as well as for the identification of radiation sources, the assessment of exposure, the survey of variations, and the principles of radiation dose distribution. Therefore, it is important to investigate human exposure sources and choose the most effective shielding methods against ionizing radiation.

Taking into account what has been reported so far, the present paper aims to evaluate the specific activity of ^238^U and ^232^Th decay chains radioisotopes and primordial ^40^K in raw building materials employed for underground parking lots through the use of high-purity germanium (HPGe) gamma-ray spectrometry. In addition, the estimation of the absorbed γ-dose rate (*D*), the annual effective dose equivalent outdoor (*AEDE_out_*) and indoor (*AEDE_in_*), the activity concentration index (*I*), and the alpha index (*I_α_*) were also accomplished with the aim to assess any possible occurrence of a radiological hazard for the population. Finally, Pearson’s correlation, a principal component analysis (PCA), and a hierarchical cluster analysis (HCA) were carried out to find out the extent of the correlations between the observed radioactivity and radiological parameters and of these parameters with the analyzed samples [29,30].

## 2. Materials and Methods

### 2.1. Description of the Samples

The investigated samples were raw building materials for underground parking lots.

A total of forty-five samples were analyzed and divided into nine groups (G#, # = 1, 2, ……, 9) (five samples per group) according to their chemical composition (Table 1).

In particular, by referring to Table 1, it is noted that (i) sample group ID G1 refers to basalt, with long-lasting friction levels in wearing courses; (ii) ID G2 refers to reclaimed asphalt pavements (RAPs), herein termed milled asphalt. It is commonly used to reduce the environmental impact of surface courses [31]; (iii) IDs G3 and G4 are red granites. Noteworthily, both basalts and granites are igneous rocks often used in surface courses, and they usually provide good performance for both abrasion and polishing; (iv) ID G5, blast-furnace slag, is a secondary aggregate (byproduct) produced in a batch process. Steel furnace slag is formed during the manufacture of steel and contains oxides and silicates. Asphalt concretes, including blast furnace slags, exhibit excellent skid resistance, surface texture, abrasion, and friction; (v) while IDs G1 and G3-G5 imply mineralogical specificity, on the other side IDs G2, G6, and G7 only provide information about production characteristics, where G2 derives from milling asphalt concretes and G6/G7 derive from crushing rocks for asphalt concrete purposes (whatever the mineralogy). In the most essential respects, this applies to the G8 and G9 IDs, where the term sand implies a size-related classification (0.063 mm to 2 mm).

All the reported materials above are usually located in the upper part of the pavement (surface courses).

### 2.2. HPGe γ-Spectrometry Analysis

For the HPGe γ-spectrometry analysis, the samples were desiccated in order to completely remove the moisture and to achieve a constant mass. After this first step, they were inserted in Marinelli hermetically sealed containers of 250 mL capacity to be uniformly spaced around the detector. After 40 days, the secular radio-active equilibrium between ^226^Ra and its daughter radionuclides was reached, and the samples were ready for γ-spectrometry counting [32]. 

Next, in order to reduce the statistical uncertainty, the samples were counted for 70,000 s, and the spectra were analyzed according to [33].

The experimental set-up consisted of a negatively biased Ortec HPGe detector (Table 2).

It was placed inside lead sumps in order to screen the background environmental radiation. Noteworthily, for the sample holder geometry of 250 mL, the efficiency and energy calibrations were performed as reported in [32]. Gamma Vision (Ortec) software was used for the data acquisition and analysis [34].

The activity concentration (Bq kg^−1^ dry weight, d.w.) of the investigated radionuclides was calculated using the following formula [35]:

(1) $$C=\frac{N_{E}}{\varepsilon_{E}t\gamma_{d}M}$$

where *N_E_* accounts for the net area of a peak at energy *E*, *ε_E_* and *γ_d_* are the efficiency and yield of the photopeak at energy *E*, respectively, *M* is the mass of analyzed samples (kg), and *t* is the live time (s). 

The measurement uncertainty is a combined standard at the coverage factor k = 2 [36]. Moreover, in order to take into account self-absorption, i.e., the phenomenon according to which there is an absorption of radiation by the matrix itself that emits it, it is important to underline that (i) for photons of an energy greater than 100 keV, self-absorption depends almost exclusively on the density of the sample; (ii) for photons of an energy less than 100 keV, it is also necessary to consider the effect of the chemical composition of the analyzed matrix. Therefore, the bulk density of the sample must be assessed and verified so that it is within the range of acceptability defined by the laboratory [37]. In cases where it is also necessary to consider the effect of the chemical composition of the sample, the same should be estimated at least approximately (also using the literature data) and verified that it is compatible with the chemical composition of the matrix used for calibration or used as input data in the application of numerical correction methods. In our case, the self-absorption corrections were carried out by using Gamma Vision software, version 8 (Ortec, Oak Ridge, TN, USA) [34].

Next, the quality of the HPGe γ-spectrometry experimental results was certified by the Italian Accreditation Body (ACCREDIA). This includes the annual continuous testing of whether the performance characteristics of the γ-spectrometry method are maintained [37]. Specifically, with reference to the blanks, they are usually ascertained on a quarterly basis after the acquisition of an unfilled lead well spectrum or an ultrapure water sample in the desired geometry specimen vessel (for naturally occurring gamma emitting radioisotopes) or, in the case of specimens that require a support medium (e.g., airborne particulate matter on a filter), through the acquisition of the clean sampling medium without changing the geometric conditions. Regarding the precision and accuracy evaluation, we followed the procedure reported below:

- Precision: the repeatability of the method was checked over time through the double-test method. A certified standard specimen (also containing ^40^K, ^226^Ra, and ^232^Th) was analyzed twice, defining as *x_1_* (the first measurement) and *x_2_* (the second measurement) the specific activity of the radionuclide of interest. The probability level *p* = 0.95 was considered. The following formula was applied:
  (2) $$\left|x_{1}-x_{2}\right|\leq \sqrt{2}\cdot s_{r}\cdot t$$
  where *t* is the Student variable and *s_r_* is the standard deviation of the repeatability obtained in the validation phase.
- Accuracy: it was assessed by comparing the certified specimen and measured values of the radionuclide of interest, taking uncertainties into account through the u-test:
  (3) $$u_{test}=\frac{measured-reference}{\sqrt{u_{meas}^{2}-u_{ref}^{2}}}\leq 2$$
  where *u_meas_^2^* and *u_ref_^2^* are the uncertainties of the measured and reference values, respectively.

### 2.3. Evaluation of the Radiological Health Risk

Several radiological parameters, such as the absorbed γ-dose rate (*D*), the annual effective dose equivalent outdoor (*AEDE_out_*) and indoor (*AEDE_in_*), the activity concentration index (*I*), and the alpha index (*I_α_*) were calculated with the aim to evaluate any possible ionizing radiation hazard for human beings [38,39]. 

#### 2.3.1. Absorbed γ-dose Rate (*D*)

According to the literature [40], the absorbed γ-dose rate (*D*) index was calculated as follows:

*D* (nGy h^−1^) = 0.462*C_Ra-226_* + 0.604*C_Th-232_* + 0.0417*C_K-40_* (4)

where *C_Ra-226_*, *C_Th-232_*, and *C_K-40_* are the mean activity concentrations of ^226^Ra, ^232^Th, and ^40^K in the investigated samples, respectively [41].

#### 2.3.2. Annual Effective Dose Equivalent Outdoor (*AEDE_out_*) and Indoor (*AEDE_in_*)

The annual effective dose equivalent for the population members was assessed through the following equations [42]:

*AEDE_out_* (mSv y^−1^) = *D* (nGy h^−1^) × 8760 h × 0.7 Sv Gy^−1^ × 0.2 × 10^−6^ (5)

*AEDE_in_* (mSv y^−1^) = *D* (nGy h^−1^) × 8760 h × 0.7 Sv Gy^−1^ × 0.8 × 10^−6^ (6)

Both values must be lower than 1 mSv y^−1^ to guarantee a negligible radiological health risk [11].

#### 2.3.3. Activity Concentration Index (*I*)

This index was defined by [11]:

*I* = *C_Ra-226_* /300 + *C_Th-232_* /200 + *C_K-40_* /3000 (7)

It describes the dose from γ-radiation existing in a building composed of a given construction material in excess of the typical external exposure. It should not be more than 1 for the radiation hazard to be neglectable.

#### 2.3.4. Alpha Index (*I_α_*)

The alpha index was evaluated as follows [43]:

*I_α_* = *C_Ra-226_* /200 (8)

It provides a measure of the exposure to indoor radon alpha radiation exhaled from building materials. The specific activity of ^226^Ra must be under 200 Bq kg^−1^ in order to avoid exposure to radon in indoor environments with activity concentrations higher than 200 Bq m^−3^ [11], and thus *I_α_* must be less than 1 in order to ensure a minimal risk of exposure to radiation [44].

### 2.4. Statistical Processing

Chemometric approaches were developed through the statistical software XLSTAT 2016 (Addinsoft, New York, NY, USA) [45]. 

In particular, a principal component analysis (PCA) was performed as a useful exploratory method according to what is reported in [46]. It is a versatile statistical method for reducing a cases-by-variable data table to its essential features, called principal components. Principal components are a few linear combinations of the original variables that maximally explain the variance of all the variables. In the process, the method provides an approximation of the original data table using only these few major components. The main graphical result is often in the form of a biplot, using the major components to map the cases and adding the original variables to support the distance interpretation of the cases’ positions. Variants of the method are also treated, such as the analysis of the grouped data [47].

Moreover, a hierarchical clusters analysis (HCA) was also used, consistent with the Ward’s algorithm, which groups specimens according to the measure of dissimilarity between them [48]. In detail, HCA is a clustering method, which explores the organization of samples in groups by subsequent data aggregations or divisions. In the case of aggregative (agglomerative) algorithms, each sample starts in its own cluster, and pairs of clusters are gradually merged into larger groups, moving up the hierarchy. Conversely, in the case of divisive algorithms, all observations start in one cluster, and splits are performed recursively, moving down the hierarchy. Hierarchical clustering solutions are typically obtained using agglomerative rather than divisive algorithms [49]. Neither PCA nor HCA generates a “mathematical model” for classification and authentication purposes. Rather, they should only be used for exploratory purposes since they display samples under investigation based on selected variables, and a natural grouping of samples may be identified [50].

## 3. Results and Discussion

### 3.1. The Specific Activity of the Radioisotopes

The mean specific activity *C_Ra-226_*, *C_Th-232_*, and *C_K-40_*, of, respectively, the ^226^Ra, ^232^Th, and ^40^K radioisotopes detected in the analyzed samples are reported in Table 3.

Across all groups of samples, it can be noticed that the activity concentration of ^226^Ra ranges between 16.5 Bq kg^−1^ d.w. (G7) and 41.2 Bq kg^−1^ d.w. (G1), with an average value of (24.2 ± 2.8) Bq kg^−1^ d.w.; that of ^232^Th varies from 1.1 Bq kg^−1^ d.w. (G7) to 53.1 Bq kg^−1^ d.w. (G1), with an average value of (26.3 ± 3.6) Bq kg^−1^ d.w; that of ^40^K ranges between 6.8 Bq kg^−1^ d.w. (G7) and 1071 Bq kg^−1^ d.w. (G4), with an average value of (445 ± 54) Bq kg^−1^ d.w.

Moreover, the specific activities of ^226^Ra, ^232^Th, and ^40^K obtained in this study for each group of samples were compared with the world average values for soil, considered as the environmental reference matrix as reported in [1]. In particular, the ^226^Ra activity concentration values were lower than that of the world average, i.e., 35 Bq kg^−1^ d.w. for all the samples except for the ID G1 group ((41.2 ± 4.8) Bq kg^−1^ d.w.). With reference to ^232^Th-specific activity, the values were lower than that of the world average, i.e., 30 Bq kg^−1^ d.w. in all cases except for the G1 ((53.1 ± 6.9) Bq kg^−1^ d.w.), G3 ((39.9 ± 5.1) Bq kg^−1^ d.w.), G4 ((42.8 ± 5.9) Bq kg^−1^ d.w.), and G9 ((35.7 ± 5.5) Bq kg^−1^ d.w.) groups. For ^40^K, the activity concentration was lower than the 420 Bq kg^−1^ d.w. average world concentration for all the analyzed samples except for the G3 ((1047 ± 116) Bq kg^−1^ d.w.), G4 ((1071 ± 127) Bq kg^−1^ d.w.), G8 ((683 ± 76) Bq kg^−1^ d.w.), and G9 ((905 ± 127) Bq kg^−1^ d.w.) groups. The different specific activities of the investigated natural radionuclides were strictly dependent on the chemical composition and mineralogical nature of the investigated samples [51]. In particular, according to the literature, the highest values of ^226^Ra, ^232^Th, and ^40^K in the sample group ID G1 (for radium), G1, G3, G4, and G9 groups (for thorium) and G3, G4, G8, and G9 groups (for potassium) could be due to the presence of ilmenites, almandine-rich garnets, and K-feldspar, respectively, as the main radioisotope-bearing minerals present in the above-reported samples [52]. Anyway, further investigations into the mineralogical composition of the analyzed samples by using analytical techniques like X-ray fluorescence (XRF) spectroscopy, micro-Raman scattering (MRS), and X-ray diffraction (XRD) will be carried out in the future.

Finally, it is worth noting that the results obtained for each group refer to the amount of natural radioactivity and not the radiation hazards to human beings. To this aim, further factors need to be taken into consideration, as stated in the section below.

### 3.2. Dose Assessment and Hazard Indices

The assessed values of the radiological hazard indices (*D, AEDE_out_, AEDE_in_, I, and I_α_*) are reported in Table 4.

D was evaluated through Equation (4), providing values ranging from 8.6 nGy h^−1^ to 79.8 nGy h^−1^, with an average value of 45.6 nGy h^−1^. The observed dissimilarities in the calculated absorbed dose values can be attributed to the different contents of natural radionuclides in the mineralogical phases of the analyzed samples [53].

In addition, Equations (5) and (6) allowed for the evaluation of *AEDE_out_* and *AEDE_in_*, respectively, providing values ranging from 10.5 µSv y^−1^ to 97.9 µSv y^−1^, with an average value of 55.9 µSv y^−1^, for *AEDE_out_*, and from 42 µSv y^−1^ to 391 µSv y^−1^, with an average value of 224 µSv y^−1^, for *AEDE_in_*, respectively. They are all under the threshold of 1 mSv y^−1^.

Moreover, the activity concentration index, provided in Equation (7), was lower than 1 for all the investigated samples, thus ensuring an almost negligible radiological health risk due to external exposure.

Finally, the value of the alpha index, as obtained using Equation (8), was found to be less than 1 in all cases, thus avoiding exposure to an indoor radon concentration of more than 200 Bq m^−3^.

### 3.3. Statistical Features

A crucial step prior to the application of any relevant statistical process consists in verifying the suitability of the normal distribution assumption of the data. For this purpose, the Bartlett test was run [54], producing a *p*-value of 0.043. Such value is lower than 0.05, thus confirming the normal distribution of the data [55]. 

Moreover, the activity concentrations of the investigated radionuclides together with the radiological indices were tested for Pearson’s correlation analysis in order to infer the interdependence and to identify any possible correlations that existed between the radiological parameters and radionuclide concentrations (Table 5).

Noteworthily, with the exception of *C_Ra-226_* and *I_α_*, all the variables showed strong positive correlations with each other. In fact, the values of all the positive correlations indicated by Pearson’s correlation matrix ranged from a minimum of 0.902 (*C_K-40_–C_Th232_*) to a maximum of 1 for the following variables: *AEDE_out_-D, D-AEDE_in_, AEDE_out_-AEDE_in_, D-I, I-AEDE_out_*, and *I-AEDE_in_*. 

Furthermore, nine variables (group IDs: *C_Ra-226_, C_Th-232_, C_K-40_, D, AEDE_out_, AEDE_in_, I*, and *I_α_*) were processed using the PCA algorithm. Table 6 shows the significant factors, i.e., principal components (PCs) extracted before the PCA elaboration.

**Figure 1:**
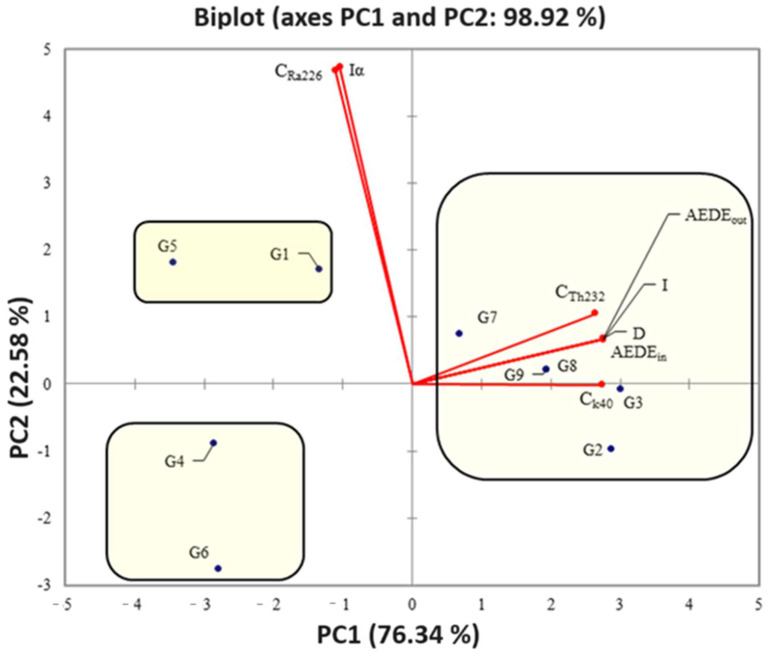
PCA biplot.

The PCA analysis results are reported in Figure 1, where the PC1 and PC2 values are highlighted, totally representing 98.92% of the total variance. 

From a first inspection of the figure, the presence of three clusters that group the analyzed samples can be identified. Moreover, the first one is composed of the G1 and G5 IDs, characterized by a positive correlation with *C_Ra-226_* and *I_α_* in terms of PC2 and a positive correlation with all variables in terms of PC1. The second one is produced by the G2, G3, G7, G8, and G9 IDs, with a positive correlation with all the variables in terms of PC1 (with the exception of *C_Ra-226_* and *I_α_*) and a positive correlation with all the variables in terms of PC2. The third cluster is composed of G4 and G6, with a positive correlation with C_Ra-226_ and I_α_ in terms of PC1, and a negative correlation with all the variables in terms of PC2. The different behavior evidenced by the PCA algorithm can be strictly dependent on the chemical composition and mineralogical nature of the investigated raw building materials for underground parking lots [56,57,58]. 

Noteworthily, G4, and G6 showed negative correlations with all the variables, and thus, they could be considered as those showing the best characteristics, the most reassuring from the radiation protection point of view, with the understanding that, as extensively reported above, all of the investigated samples appeared to be nonhazardous to humans.

**Figure 2:**
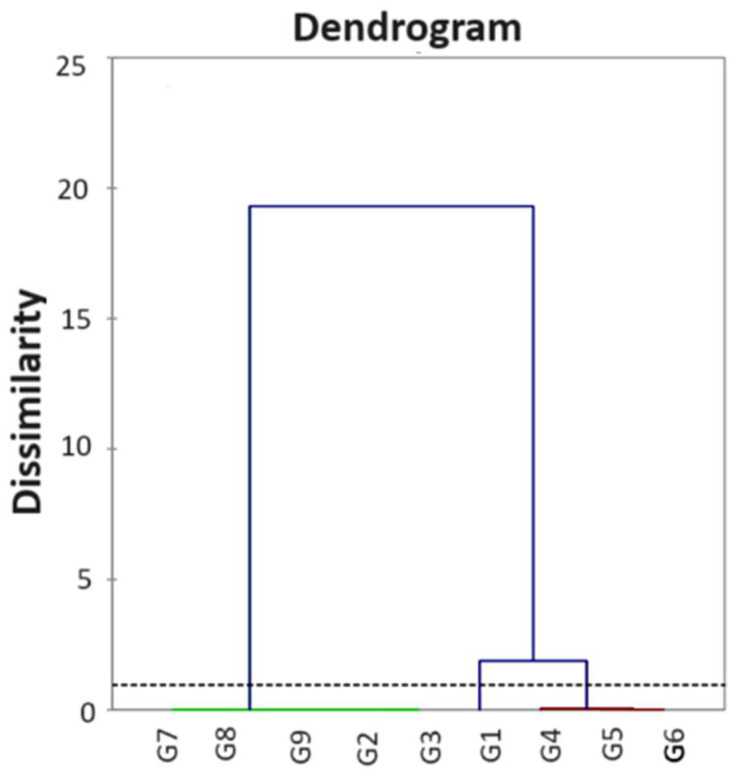
Dendrogram reporting the HCA statistical results.

Finally, with reference to the HCA, the outcome dendrogram is shown in Figure 2.

**Figure 3:**
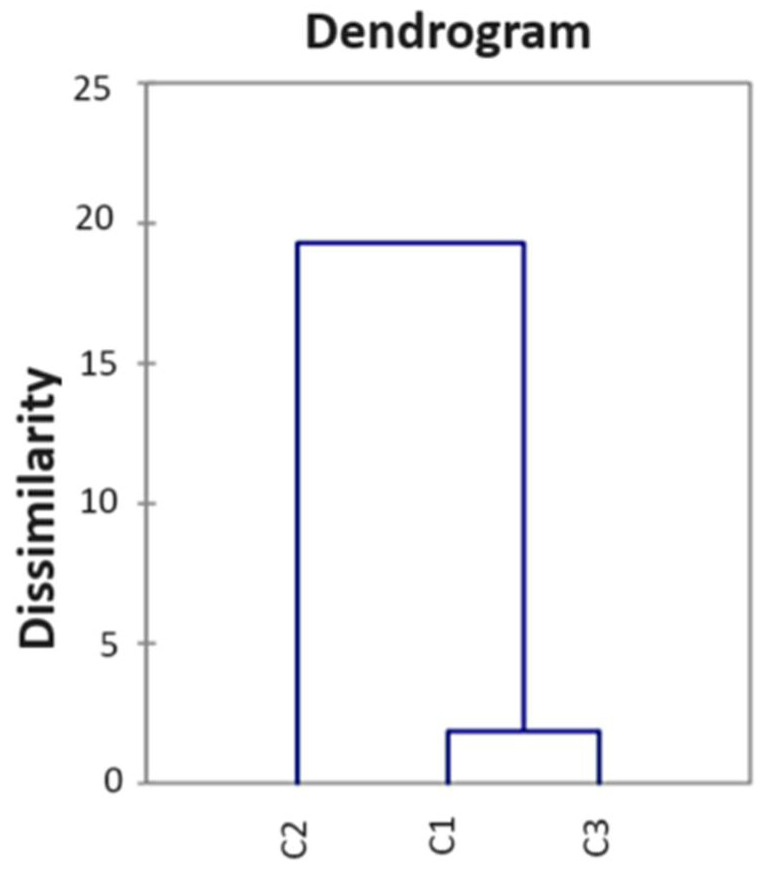
HCA statistical results after a cut at 0.954.

The automatic cut (dotted line) was placed on the dendrogram at a 0.954 distance, and it implied the formation of three clusters. In detail, the first cluster regrouped G1 and G5, whereas G2, G3, G7, G8, and G9 fell into the second cluster, and G4 and G6 fell into the third cluster. Noteworthily, from the HCA, it appeared that the samples were grouped into three different clusters (C1, C2, and C3) (Figure 3) in very good agreement with the results provided by the PCA. 

It is worth noting that the three groups of materials identified in this work correspond to the generally accepted grouping of natural materials according to their chemical composition and mineralogical nature [59,60].

## 4. Conclusions

The natural radioactivity content of the raw building materials used for underground parking lots (samples G1–G10) was investigated through high-purity germanium (HPGe) γ-ray spectrometry. In particular, the activity concentration of ^226^Ra ranged from 16.5 Bq kg^−1^ d.w. (G7) to 41.2 Bq kg^−1^ d.w. (G1), with an average value of (24.2 ± 2.8) Bq kg^−1^ d.w.; that of ^232^Th varied from 1.1 Bq kg^−1^ d.w. (G7) to 53.1 Bq kg^−1^ d.w. (G1), with an average value of (26.3 ± 3.6) Bq kg^−1^ d.w; that of ^40^K ranged between 6.8 Bq kg^−1^ d.w. (G7) and 1071 Bq kg^−1^ d.w. (G4), with an average value of (445 ± 54) Bq kg^−1^ d.w. The different specific activities of ^226^Ra, ^232^Th, and ^40^K, detected in the analyzed samples were strictly dependent on their chemical composition and mineralogical nature.

Moreover, calculations of the absorbed γ-dose rate (*D*), the annual effective dose equivalent outdoor (*AEDE_out_*) and indoor (*AEDE_in_*), the activity concentration index (*I*), and the alpha index (*I_α_*) were carried out with the aim to evaluate any possible ionizing radiation hazards for human beings. The obtained values were all lower than the threshold ones for the public, i.e., 1 mSv y^−1^ for *AEDE_out_* and *AEDE_in_* and 1 for *I* and *I_α_*, respectively, thus reasonably ruling out radiological hazard effects.

Finally, Pearson’s correlation and principal component and hierarchical cluster multivariate statistical analyses were carried out to determine the correlations between the observed radioactivity and radiological parameters and with the analyzed samples, providing the following main findings: (i) all the investigated variables could be grouped into three clusters, and the three groups of materials identified in this work corresponded to the generally accepted grouping of natural materials according to their chemical composition and mineralogical nature; (ii) G4 and G6 showed negative correlations with all the variables, and thus they could be considered as those showing the best characteristics, the most reassuring from the radiation protection point of view, with the understanding that, as extensively reported in the paper, all of the investigated samples appear to be nonhazardous to humans.

## Figures and Tables

**Table 1:** The investigated raw materials together with their identification code (Group ID) and number of analyzed samples for each group.

Group ID	Raw Material	Number of Samples
G1	Basalt	5
G2	Milled asphalt	5
G3	Fine red granite	5
G4	Coarse red granite	5
G5	Blast-furnace slag	5
G6	Crushed stone	5
G7	Gravel	5
G8	Yellow sand	5
G9	Grey sand	5

**Table 2:** The HPGe detector operating parameters.

HPGe Detector	
FWHM	1.94 keV
Peak-to-Compton ratio	65:1
Relative efficiency	37.5% (at the 1.33 MeV ^60^Co γ-line)
Bias voltage	−4800 V
Energy range	5 keV–2 MeV

**Table 3:** The mean specific activity *C_Ra-226_*, *C_Th-232_*, and *C_K-40_*, of, respectively, ^226^Ra, ^232^Th, and ^40^K (Bq kg^−1^ d.w.), evaluated for the investigated groups of samples.

Group ID	*C_Ra-226_* (Bq kg^−1^ d.w.)	*C_Th-232_* (Bq kg^−1^ d.w.)	*C_K-40_* (Bq kg^−1^ d.w.)
G1	41.2 ± 4.8	53.1 ± 6.9	157 ± 19
G2	27.2 ± 2.9	29.5 ± 3.8	113 ± 14
G3	17.3 ± 1.9	39.9 ± 5.1	1047 ± 116
G4	20.1 ± 2.6	42.8 ± 5.9	1071 ± 127
G5	21.1 ± 1.9	4.4 ± 1.1	9.9 ± 1.3
G6	29.2 ± 4.8	1.3 ± 0.5	12.7 ± 5.6
G7	16.5 ± 0.6	1.1 ± 0.4	6.8 ± 2.7
G8	23.8 ± 2.6	28.7 ± 3.7	683 ± 76
G9	21.2 ± 3.1	35.7 ± 5.5	905 ± 127
Average	24.2 ± 2.8	26.3 ± 3.6	445 ± 54

**Table 4:** Radiological hazard indices.

Group ID	*D* (nGy h^−1^)	*AEDE_out_* (µSv y^−1^)	*AEDE_in_* (µSv y^−1^)	*I*	*I* *_α_*
G1	57.7	70.7	282	0.46	0.21
G2	35.1	43.0	172	0.28	0.14
G3	75.8	92.9	371	0.61	0.09
G4	79.8	97.9	391	0.64	0.10
G5	12.8	15.7	62	0.10	0.11
G6	14.8	18.2	72	0.11	0.15
G7	8.6	10.5	42	0.06	0.08
G8	56.8	69.7	278	0.45	0.12
G9	69.1	84.7	339	0.55	0.11
Average	45.6	55.9	224	0.4	0.10

**Table 5:** Pearson’s correlation matrix.

Variables	*C_Ra-226_*	*C_Th-232_*	*C_K-40_*	*D*	*AEDE_out_*	*AEDE_in_*	*I*	*I_α_*
*C_Ra-226_*	1							
*C_Th-232_*	−0.195	1						
*C_K-40_*	−0.393	0.902	1					
*D*	−0.274	0.963	0.982	1				
*AEDE_out_*	−0.273	0.963	0.982	1.000	1			
*AEDE_in_*	−0.274	0.963	0.982	1.000	1.000	1		
*I*	−0.275	0.964	0.982	1.000	1.000	1.000	1	
*I_α_*	0.995	−0.171	−0.370	−0.250	−0.249	−0.250	0.251	1

**Table 6:** Significant factors extracted before the PCA elaboration.

PC1	PC2	PC3
6.107	1.806	0.082
76.336	22.580	1.025
76.336	98.916	99.941

## Data Availability

Data are contained within the article.
